# PEDV-spike-protein-expressing mRNA vaccine protects piglets against PEDV challenge

**DOI:** 10.1128/mbio.02958-23

**Published:** 2024-01-17

**Authors:** Yongxiang Zhao, Baochao Fan, Xu Song, Jie Gao, Rongli Guo, Cheng Yi, Zhaoming He, Hongpeng Hu, Jianhao Jiang, Lixiang Zhao, Tianyi Zhong, Bin Li

**Affiliations:** 1 Institute of Veterinary Medicine, Jiangsu Academy of Agricultural Sciences, Nanjing, China; 2 Key Laboratory of Veterinary Biological Engineering and Technology, Ministry of Agriculture, Nanjing, China; 3 Jiangsu Key Laboratory for Food Quality and Safety-State Key Laboratory Cultivation Base of Ministry of Science and Technology, Nanjing, China; 4 Jiangsu Co-innovation Center for Prevention and Control of Important Animal Infectious Diseases and Zoonoses, Yangzhou, China; 5 GuoTai (Taizhou) Center of Technology Innovation for Veterinary Biologicals, Taizhou, China; 6 Suzhou Huiliao Biomedical Technology Co. Ltd., Suzhou, China; 7 Suzhou Medical College, Soochow University, Suzhou, China; University of Calgary, Calgary, Canada

**Keywords:** mRNA vaccine, PEDV, spike protein, protective immunity, piglets

## Abstract

Porcine epidemic diarrhea virus (PEDV), a swine enteropathogenic coronavirus, causes severe diarrhea in neonatal piglets, which is associated with a high mortality rate. Thus, developing effective and safe vaccines remains a top priority for controlling PEDV infection. Here, we designed two lipid nanoparticle (LNP)-encapsulated mRNA (mRNA-LNP) vaccines encoding either the full-length PEDV spike (S) protein or a multiepitope chimeric spike (Sm) protein. We found that the S mRNA-LNP vaccine was superior to the Sm mRNA-LNP vaccine at inducing antibody and cellular immune responses in mice. Evaluation of the immunogenicity and efficacy of the S mRNA vaccine in piglets confirmed that it induced robust PEDV-specific humoral and cellular immune responses *in vivo*. Importantly, the S mRNA-LNP vaccine not only protected actively immunized piglets against PEDV but also equipped neonatal piglets with effective passive anti-PEDV immunity in the form of colostrum-derived antibodies after the immunization of sows. Our findings suggest that the PEDV-S mRNA-LNP vaccine is a promising candidate for combating PEDV infection.

**IMPORTANCE:**

Porcine epidemic diarrhea virus (PEDV) continues to harm the global swine industry. It is important to develop a highly effective vaccine to control PEDV infection. Here, we report a PEDV spike (S) mRNA vaccine that primes a potent antibody response and antigen-specific T-cell responses in immunized piglets. Active and passive immunization can protect piglets against PED following the virus challenge. This study highlights the efficiency of the PEDV-S mRNA vaccine and represents a viable approach for developing an efficient PEDV vaccine.

## INTRODUCTION

Porcine epidemic diarrhea virus (PEDV), the main causative agent of porcine epidemic diarrhea (PED), causes a severe enteric disease characterized by diarrhea, vomiting, and dehydration. Newborn piglets are especially susceptible to PEDV, which can induce mortality rates of up to 100% (1). PEDV was first identified in Europe in the early 1970s. Since 2010, highly pathogenic variant PEDV strains have gradually infiltrated the swine population and caused substantial economic losses worldwide. PEDV continues to be a major problem in the swine industry to date (2–5). Unfortunately, most currently available conventional inactivated and attenuated vaccines are not sufficiently effective at controlling PEDV or have an inadequate safety profile (6). Therefore, the development of novel protective vaccines remains a top priority for the control of PED.

PEDV is a positive-sense, single-stranded, enveloped RNA virus belonging to the *Coronaviridae* family. The PEDV genome consists of seven open reading frames (ORFs), which encode 16 nonstructural proteins (Nsp1-16) and 4 structural proteins [spike (S), envelope, membrane, and nucleocapsid] (7, 8). Among these viral proteins, the S glycoprotein on the virion surface plays a critical role in the interaction with the host, which is responsible for host cell receptor binding and the subsequent fusion with the host cell membrane (9, 10). Because the S protein contains multiple neutralizing epitopes, the major target of neutralizing antibodies, it is the primary immunogen and an ideal antigenic target for vaccine development (11–14).

Messenger RNA (mRNA)-based vaccines have demonstrated great promise in the fight against viral diseases caused by pathogens such as SARS-CoV-2, influenza virus, Zika virus, Dengue virus, Hepatitis C virus, and HIV (15–20). The mRNA vaccine also shows good prospects in preventing and controlling bacterial and spirochetal infections (21–23). These mRNA vaccines display strong immunogenicity and high efficacy. The mRNA platform permits the rapid generation of vaccines against multiple targets while also acting as a self-adjuvant to stimulate the innate immune system (24, 25). We, therefore, selected this system to develop a PEDV mRNA vaccine.

In this study, we designed two nucleoside-modified mRNA vaccines targeting the S glycoprotein of PEDV, one encoding the full-length S protein and the other encoding a multiepitope chimeric spike protein (Sm), and then encapsulated them in lipid nanoparticles (LNPs). We investigated the ability of these mRNA-LNP vaccines to induce PEDV-specific antibodies and cellular immune response in mice and pigs before evaluating their protective efficacy against PEDV in piglets. Our results demonstrated that the full-length S mRNA vaccine induced better immunity than the Sm mRNA vaccine and that the S mRNA vaccine protected actively and passively immunized piglets against PEDV.

## MATERIALS AND METHODS

### mRNA production and LNP encapsulation

The S glycoprotein of the AH2012/12 strain of PEDV (GenBank: KC210145) was employed as the reference amino acid sequence. Two antigens, the S and Sm proteins, were selected for mRNA vaccine design. The Sm protein was composed of the N terminal domain (NTD, 19–233 aa), the collagenase equivalent domain (COE, 499–638 aa), and several linear neutralizing epitopes (744–774 aa) (26). The mRNA was synthesized by *in vitro* transcription using the T7 RNA polymerase and a linearized plasmid DNA template containing the optimal codons of the S glycoprotein.

Lipids were dissolved in ethanol containing an ionizable lipid, 1,2-distearoyl-sn-glycero-3-phosphocholine (DSPC), PEG lipid, and cholesterol. The lipid mixture was combined with 10-mM citrate buffer (pH 4.0) containing mRNA at a ratio of 1:3 (ethanol to aqueous fraction) using a microfluidic mixer (Micro & Nano Technology, China). Formulations were dialyzed against phosphate buffer saline (PBS) (pH 7.4) and then concentrated using Amicon ultra centrifugal filters (Millipore, USA) with a 10-kD molecular weight cut-off. All formulations were tested for particle size and polymer dispersity index (PDI).

### Biodistribution and expression of mRNA encoding the PEDV S glycoprotein

LNPs containing 5 µg of Firefly luciferase (FLuc)-tagged S or Sm mRNA were introduced via intramuscular injection into BALB/c mice, and the mice were injected with PBS as negative controls. After 6 h, mice were anesthetized with isoflurane gas and administered 3 mg of D-luciferin (dissolved in PBS) via intraperitoneal injection. Images were captured 10 min after luciferin injection using an *in vivo* imaging system (PerkinElmer, USA). Subsequently, mice were sacrificed, and their organs were collected for *ex vivo* imaging. The fluorescence intensity was detected, and the data were expressed as photon flux.

To detect the expression of LNP-delivered mRNA, HEK293T cells were seeded into 24-well plates at 200,000 cells/well. After 18 h, the cells were treated with LNPs containing S or Sm mRNA (1 µg per well), followed by another 24 h incubation. The untreated cells served as the control group. The expression of mRNA was detected by indirect immunofluorescence (IF) and western blotting assays using our laboratory’s primary anti-PEDV-S monoclonal antibody.

### Design of the mouse vaccination experiments

The immunization schedule is shown in Fig. 2A. In the mouse immunization experiments, 15 BALB/c mice (6 weeks old) were randomly assigned to three groups of five mice per group. Two groups of mice received a dorsal subcutaneous injection of mRNA vaccine (either S or Sm) at a dose of 30 µg per mouse. Control mice received a dorsal subcutaneous injection of PBS. The vaccinated mice also received a booster dose on day 14 post-vaccination (dpv). Blood was collected from each mouse before vaccination and at 14 and 28 dpv. The serum was separated for antibody detection. At 28 dpv, the mice were sacrificed, and their splenic lymphocytes were isolated to analyze the cellular immune response.

### Design of the pig vaccination experiments and viral challenge study

In the active immunization and challenge experiments, 22 1-d-old, PEDV-naive piglets were randomly assigned to one of two groups (11 piglets per group) and housed in separate rooms. The piglets in the treatment group were injected into the neck muscles with the S mRNA vaccine (45 µg per piglet), while the piglets in the control group were injected with PBS. The vaccinated piglets also received a booster dose at 14 dpv. Serum samples were collected from each piglet prior to vaccination and at 14, 28, 60, 90, and 120 dpv. At 28 dpv, six piglets were randomly selected from each group for the viral challenge experiments. The piglets were challenged with the AH2012/12 strain of PEDV via the oral route of inoculation (2.0 × 10^7.0^ TCID_50_ per piglet). After the viral challenge, fecal swab samples and fecal consistency scores were collected daily. At 4 and 11 d post-challenge (dpc), two and four pigs, respectively, were killed and necropsied. Intestinal tissue and content samples were collected for pathological evaluation and quantification of PEDV RNA load, respectively.

In the passive immunization and challenge experiments, two PEDV-naive pregnant sows were injected into the neck muscles with the S mRNA vaccine (100 µg per sow) or PBS 1 month before parturition. The vaccinated sows also received a booster dose of vaccine 14 d later. On the day of farrowing, serum and colostrum samples were collected from each sow for antibody detection. After being allowed to suckle for 5 d, five newborn piglets were randomly selected from each sow and challenged with the AH2012/12 strain of PEDV via the oral route of inoculation (2 × 10^6.0^ TCID_50_ per piglet). After the viral challenge, fecal swab samples and fecal consistency scores were collected daily. At 3 and 10 dpc, two and three piglets, respectively, were killed and necropsied. Intestinal tissue and content samples were collected for pathological evaluation and quantification of PEDV RNA load, respectively.

### ELISA for detecting PEDV-S-specific antibodies

The enzyme-linked immunosorbent assay (ELISA) for PEDV-S binding IgG and IgA detection was performed as previously reported (27). Briefly, wells of a 96-microtiter plate were coated with recombinant eukaryotic PEDV-S1 or PEDV-Sm protein (100 ng/well), suspended in bicarbonate buffer (100 mM, pH 9.6), and incubated at 4°C overnight. After discarding the coating solution, the microplates were washed three times with PBST buffer (PBS supplemented with 0.05% Tween 20, pH 7.4) and blocked with 3% BSA at 37°C for 2 h. After another washing step, the diluted serum or colostrum samples (1:100 in blocking buffer) were applied to the plate. PEDV hyperimmune pig serum was used as a positive control, while PEDV-naive pig serum was used as a negative control. After incubation at 37°C for 30 min, the plates were washed three times with PBST buffer. Then, 100 µL of Goat anti-pig IgG or Goat anti-pig IgA [both horseradish peroxidase (HRP)-conjugated] was added to each well and incubated for 30 min at 37°C. Subsequently, 100 µL of the TMB substrate solution was added to each well, followed by a 15-min incubation at 37°C. Finally, 50 µL of H_2_SO_4_ (2 M) was added per well to stop the reaction and absorbance at 450 nm optical density 450 (OD450) was measured.

### Neutralization assays

The collected serum samples were heat inactivated for 30 min at 56°C and serially diluted in twofold increments. The diluted samples were then mixed with an equal PEDV volume (200 TCID_50_) and incubated at 37°C for 1 h. Subsequently, 0.1 mL of each mixture was transferred to a Vero (monkey kidney) cell monolayer, which was cultured in a 96-well tissue culture plate and washed once with Dulbecco’s modified Eagle medium (DMEM, Gibco, USA) before PEDV application. After adsorption for 1.5 h at 37°C, the inoculate was discarded and the cells were washed twice with PBS. Next, the maintenance medium containing trypsin (10 µg/mL) was added to each well and the plate was incubated for 48 h at 37°C. Cells were examined daily for cytopathic effects (CPE). The neutralizing antibody titers were expressed as the highest serum dilution that protected more than 50% of the cells from CPE. The PEDV strains AH2012/12 (GenBank: KC210145), HK2021 (GenBank: OL762457), and JS2008 (GenBank: KC109141) were isolated in our laboratory.

### Flow cytometry

At 28 dpv, the splenic lymphocytes of mice or the peripheral blood lymphocytes of piglets were isolated, transferred into a 1.5-mL centrifuge tube (1 × 10^6^ cells/tube), and washed once with PBS. The pellet was resuspended in 300 µL of cell fluorescence solution (Flow Cytometry Staining Buffer, eBioscience, USA) and stained with the following fluorescent antibodies: FITC hamster anti-mouse CD3e (BD Biosciences, USA), PE anti-mouse CD4 (BioLegend, USA), APC anti-mouse CD8a (BioLegend), FITC mouse anti-pig CD3ε (BD Biosciences), PE mouse anti-pig CD4a (BD Biosciences), and APC mouse anti-pig CD8α (SouthernBiotech, USA), at room temperature in the dark for 30 min. The tubes were centrifuged at 1,500 rpm for 5 min; the supernatant was removed, and the pellet was washed twice with PBS. The cell pellet was resuspended in 500 µL of fluorescence preservation solution (0.15 M PBS pH 7.4, 2% glucose, 1% formaldehyde, and 0.1% NaN_3_). Flow cytometry, performed on a Accuri C6 Plus instrument (BD Biosciences), was then used to enumerate CD4^+^ and CD8^+^ T cells per 1 × 10^5^ cells acquired. Data analysis was performed using FlowJo v10.7.1.

### Lymphocyte proliferation assay

At 28 dpv, mouse splenic lymphocytes or piglet peripheral blood lymphocytes were isolated for use in the lymphocyte proliferation assay. In brief, 100 µL of the cell suspension (5 × 10^6^ cells/mL) was applied to each well of a 96-well plate and stimulated with 10 µg/mL of recombinant eukaryotic PEDV-S protein. Six replicate wells were set up per sample. The 96-well cell culture plate was placed in a 5% CO_2_ incubator at 37°C for ~72 h. The cell culture supernatant was collected for the cytokine detection assay and replaced with a fresh medium. Next, 10 µL of CCK-8 solution (Beyotime, China) was added to each well. After a 2-h incubation at 37°C, the absorbance at 490 nm (OD490) was measured. The relative stimulation index was calculated as the ratio of the average OD value of antigen-stimulated wells to that of unstimulated wells.

### IL-4 and IFN-γ detection

The cell culture supernatants (from the abovementioned lymphocyte proliferation assay) were collected after ~72 h of stimulation with the recombinant eukaryotic PEDV-S protein. The IL-4 and IFN-γ levels in the supernatants were evaluated by using commercial Swine IL-4 and IFN-γ ELISA Kits (Meimian, China) according to the manufacturer’s instructions. The cytokine concentrations were calculated according to the standard curve obtained for each ELISA plate.

### Evaluation of clinical signs and gross/histological lesions

After the viral challenge, pigs were monitored daily for clinical signs of diarrhea. Diarrhea was assessed by scoring fecal consistency. The scores ranged from 0 to 3, with 0 being normal solid feces, 1 being pasty feces, 2 being semiliquid diarrhea with some solid content, and 3 being liquid diarrhea with no solid content. Piglets with scores of 2 or more were considered diarrheic. At necropsy, the jejunum and ileum tissues were collected and fixed in formalin for histological examination. The dehydrated tissues were treated with xylene, embedded in paraffin wax, sliced, and mounted on slides. The slides were subjected to H&E-stained and immunohistochemistry (IHC) assay. The monoclonal antibody against the PEDV N protein (made in our laboratory) was used as the primary antibody (28), and an Alexa Fluor 555-labeled Donkey anti-mouse IgG (Beyotime) was used as the secondary antibody to perform IHC.

### Quantification of PEDV RNA by quantitative real-time PCR

The number of viral RNA copies in fecal swabs and tissue samples was determined by TaqMan quantitative real-time (qRT)-PCR. Viral RNA was extracted from 10% of the supernatant obtained from fecal swabs or homogenized intestinal tissue samples. The viral RNA was reverse transcribed into cDNA using a Reverse Transcription Kit (Vazyme, China). cDNA amplification was performed using the Taq Pro HS Probe Master Mix (Vazyme) according to the manufacturer’s instructions. The following *PEDV-N* gene primer and probe sequences were used: sense, 5′-GTCTGAAAAGCCAATCATTC-3′; antisense, 5′-TTGCCTCTGTTGTTACTC-3′; and probe 5′-CTGTTGTTGCCATTGCCACGA-3′.

### Western blotting

To detect antigen expression following mRNA-LNP vaccination, 293T cells cultured in 24-well plates were incubated with the mRNA-LNP vaccine (1 µg per well) for 24 h. The cells were washed with cold PBS and lysed with RIPA lysis buffer (Beyotime). Equal amounts of protein were subjected to SDS-PAGE and electrotransferred onto nitrocellulose membranes (Pall, USA). The membranes were blocked in 5% nonfat milk for 2 h at room temperature and then incubated with an anti-PEDV-S monoclonal antibody (made in our laboratory). After three washes with PBST, the membranes were incubated with an HRP-conjugated goat anti-mouse antibody (Beyotime) for 1 h at room temperature. After washing, proteins were detected using an ECL kit (Tanon, China).

### Indirect immunofluorescence assay

The 293T cells were treated with mRNA-containing LNPs, washed three times with PBS, fixed for 20 min with 4% paraformaldehyde, and then permeabilized for 30 min with 0.1% Triton X-100. The fixed cells were incubated with an anti-PEDV-S monoclonal antibody for 2 h at 37°C. After three washes with PBS, the cells were incubated with an Alexa-Fluor-488-conjugated goat anti-mouse IgG (Beyotime) for 1 h at 37°C. The cell nuclei were stained with 0.01% 4′,6-diamidino-2-phenylindole (DAPI) for 10 min at room temperature. After washing again with PBS, fluorescence images were observed using a fluorescence microscope (Nikon, Japan).

### Statistical analysis

GraphPad Prism 8 software was used for statistical analysis and figure generation. All data were expressed as the mean ± SD. Differences between groups were examined for statistical significance using a mixed-effects analysis or a one-way analysis of variance (ANOVA) with Tukey’s multiple comparison post-test. The asterisks in the figures indicate significant differences, with *P* < 0.05 (*P < 0.05; **P < 0.01; ns, not significant).

## RESULTS

### Production and characteristics of PEDV-S-based mRNA vaccines

**Fig 1:**
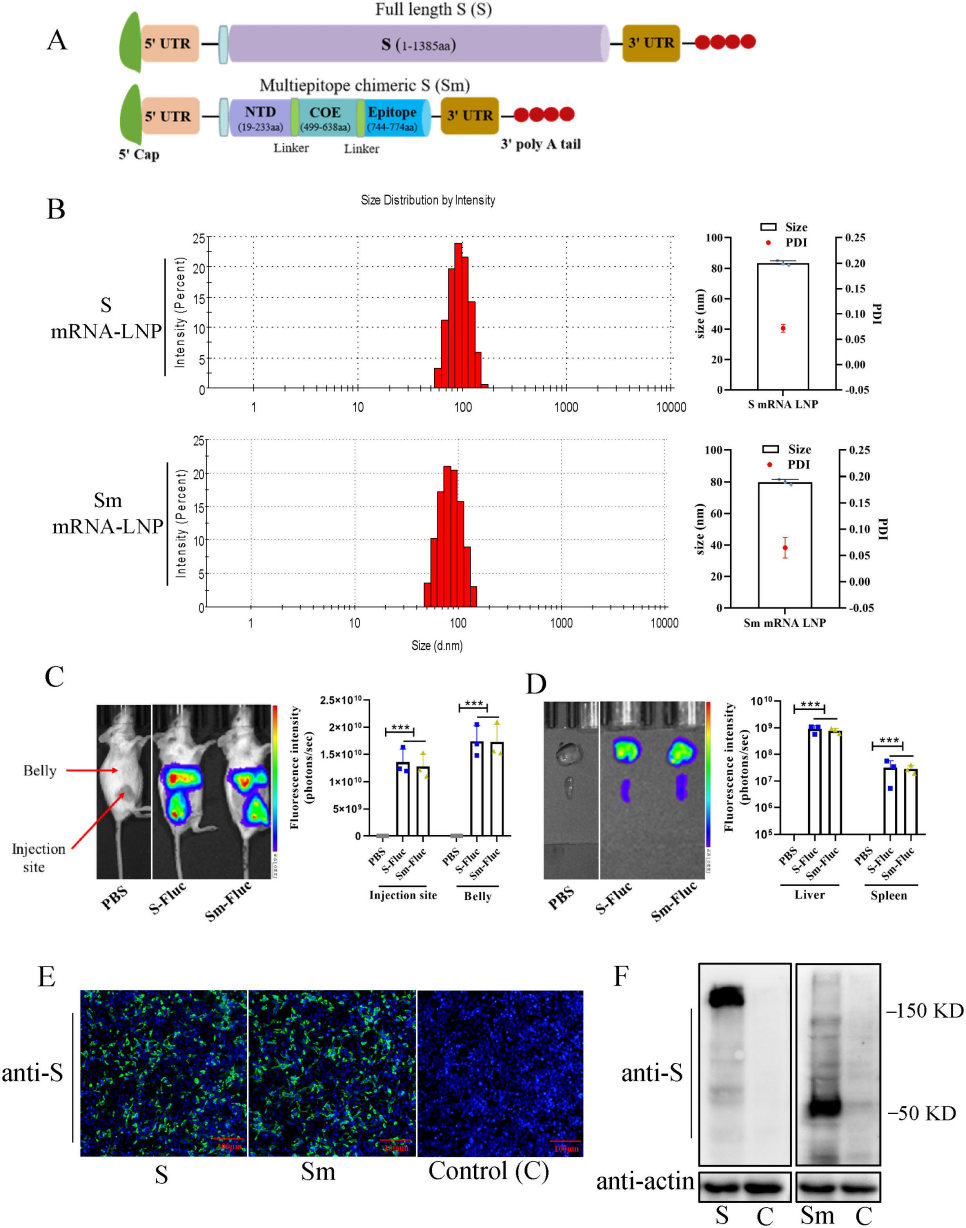
Production and characteristics of PEDV-S-based mRNA vaccines. (**A**) Illustration of mRNA constructs, which include the full-length S protein and a multiepitope chimeric spike protein composed of the N terminal domain, collagenase equivalent domain, and several linear neutralizing epitopes. (**B**) Representative particle size and polymer dispersity index graph of mRNA-LNPs. The particle size and PDI of mRNA-LNPs were tested using dynamic light scattering on a NS-90Z (OMEC, China). mRNA-LNPs were irradiated with a red laser (λ = 633 nm), and scattered light was detected at a backscattering angle of 90°. Results were analyzed using the software (Dylisizer Software 2.00). (**C**) Biodistribution of the mRNA-LNPs. Mice were administrated intramuscularly with luciferase-tagged mRNA-LNP. Six hours later, mice were anesthetized, along with the intraperitoneal injection of D-luciferin Bioluminescence. Images were captured using *In Vivo* Imaging System, and the fluorescence intensity was measured. (**D**) Major organ distribution of the mRNA-LNPs in mice. Mice were sacrificed, and organs were collected for *ex vivo* imaging and fluorescence intensity detection. (**E and F**) Expression of mRNA-LNPs *in vitro*. 293T cells were treated with mRNA-containing LNPs for 24 h, then fixed for IFA (**E**), or lysed for western blotting (**F**). The anti-PEDV-S monoclonal antibody was the primary antibody for the IFA and western blotting assay. PEDV S was labeled with green fluorescence, and cell nuclei (blue) were stained with DAPI.

Two PEDV-S-protein-based mRNA vaccines were designed: one vaccine expressed the full-length S protein and the other expressed the Sm protein, composed of an NTD (19–233 aa), a COE (499–638 aa), and several linear neutralizing epitopes (744–774 aa). To efficiently express the target protein, we designed a modified mRNA encoding a type 1 (N7mGpppAm) cap, 5′ and 3′ untranslated sequences, and an optimized target antigen S or Sm sequence (Fig. 1A). The S and Sm mRNA transcripts were then encapsulated in LNPs. The resulting mRNA-LNPs had an average particle size of 83.38 nm and a narrow distribution with a PDI of 0.072, with over 95% encapsulation efficiency (Fig. 1B). The PDI < 0.1 indicated that the mRNA-LNP particles were more uniform size, and the size of 83 nm is one of the optimal sizes that facilitate their internalization and biodistribution. To visualize the tissue distribution of mRNA-LNPs, mRNAs were tagged with the FLuc reporter. The FLuc-tagged S and Sm mRNA-LNPs were then intramuscularly injected into BALB/c mice. There was no fluorescence detected in control mice. Notably, the mRNA-LNPs were efficiently delivered *in vivo* and induced strong FLuc expression at the injection site and in the mouse belly 6 h post-injection (Fig. 1C). Further *ex vivo* dissection showed a robust accumulation of mRNA-LNPs in the spleen and liver (Fig. 1D). *In vitro* immunogen expression was verified by detection of the S and Sm proteins in the mRNA-transfected 293T cells. The indirect immunofluorescence assay (IFA) (Fig. 1E) and western blotting (Fig. 1F) results showed that incubation of 293T cells with mRNA-LNPs resulted in the efficient expression of the S and Sm proteins.

### Immunogenicity of S versus Sm mRNA vaccines in mice

**Fig 2:**
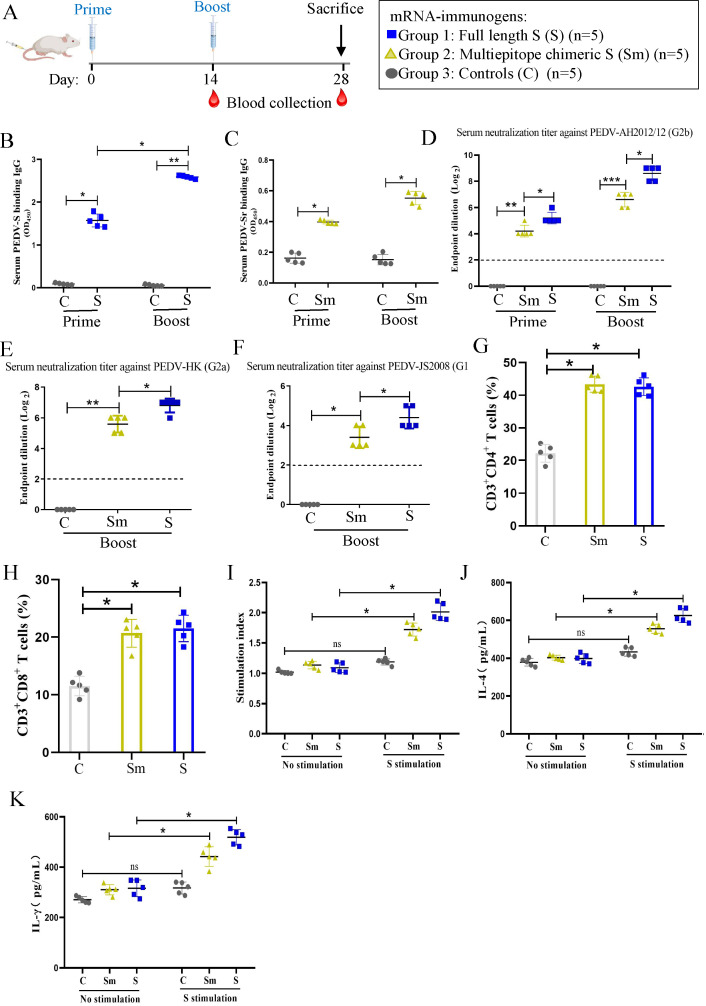
Immunogenicity of S versus Sm mRNA vaccines in mice. (**A**) Experimental design for the mice vaccination. Mice were immunized at day 0 and day 14, with mRNA either expressing the full-length S (group 1) or a multiepitope chimeric spike protein (Sm) (group 2). A third group was inoculated with PBS as a control (group 3). (**B and C**) The levels of PEDV S and Sm protein binding IgG and IgA antibodies in serum were measured at 14 and 28 d after the primary immunization. (**D–F**) The serum-neutralizing antibodies against different PEDV strains were detected, including G2b strain AH2012/12 (**D**), G2a strain HK (**E**), and G1 strain JS2008 (**F**). The neutralizing antibody titers were expressed as the highest serum dilution that protected more than 50% of the cells from CPE. The dotted line indicates the lower limit of detection. (**G and H**) The frequencies of CD3^+^CD4^+^ and CD3^+^CD8^+^ T cells in mice after immunization. The splenic lymphocytes of mice were isolated at 28 dpv and characterized by flow cytometry to enumerate CD4^+^ and CD8^+^ T cells per 1 × 10^5^ cells acquired. Data analysis was performed using FlowJo v10.7.1. (**I**) PEDV S-specific lymphocyte proliferation assay. The lymphocytes were stimulated with S protein for 72 h, and the proliferation rate was measured using a CCK-8 kit. The relative stimulation index was calculated as the ratio of the average OD value of antigen-stimulated wells to that of unstimulated wells. (**J–K**) IL-4 and IFN-γ secretion of lymphocytes after re-stimulation with S protein. The cell culture supernatant was collected for the IL-4 and IFN-γ detection by ELISA. Data are presented as the mean ± SD of five mice per group. The asterisks in the figures indicate significant differences; *P* < 0.05 represents a statistically significant difference (*P < 0.05; **P < 0.01; ns, not significant).

We next compared the immunogenicity of PEDV S with Sm mRNA vaccines in a mouse model. The study design, which included three groups of mice, is summarized in Fig. 2: group 1 was inoculated with S mRNA, group 2 with Sm mRNA, and group 3 with PBS as a control. All the mice in groups 1 and 2 received two inoculations 14 d apart. Blood was collected from mice at 2-week intervals until d 28. The mice experienced no adverse events after vaccination. The two mRNA vaccines were immunogenic, as evidenced by the appearance of anti-PEDV-S and Sm antibodies after the primary immunization (priming); moreover, the titers of these antibodies were markedly increased after the second immunization (boosting) (Fig. 2B and C). However, the mean binding antibody titers in the S group were higher than those in the Sm group after priming and boosting. Neutralizing antibodies against different PEDV strain subtypes were also detected. The mean neutralizing antibody titers against the PEDV G2b AH2012/12 (Fig. 2D), the G2a HK2021 (Fig. 2E), and the G1 JS2008 (Fig. 2F) strains were higher in the S group than in the Sm group after priming or boosting; no neutralizing antibodies were detected in the control group. Moreover, the level of neutralization against the homologous G2b strain was the highest among all the strains targeted, while the neutralization ability of antibodies against the heterogeneous G1 strain was the weakest.

To compare cellular immune responses induced by the two mRNA vaccines, mouse splenic lymphocytes were isolated at 28 dpv and subjected to T lymphocyte subset characterization and enumeration by flow cytometry. The results showed that the percentages of CD3^+^CD4^+^ and CD3^+^CD8^+^ T cells in both the S and Sm mRNA groups were higher than that in the PBS control group (Fig. 2G and H). The lymphocytes were restimulated *in vitro* with a recombinant eukaryotic PEDV-S protein (10 µg/mL) to analyze antigen-specific lymphocyte proliferative responses. The stimulation indices of the S and Sm mRNA immunization groups were significantly higher than that of the control group, with the proliferative rate of the S group being higher than that of the Sm group (Fig. 2I). To further characterize the cellular immune responses induced by the mRNA vaccines, IFN-γ and IL-4 secretion by lymphocytes restimulated with the S protein was measured by ELISA. We found that the lymphocytes from S-mRNA-vaccinated mice produced significantly higher levels of IFN-γ and IL-4 than those from control mice; moreover, the S group lymphocytes exhibited higher cytokine production than those from the Sm group (Fig. 2J and K). These data indicate that the S mRNA is superior to the Sm mRNA at inducing both humoral and cellular immune responses.

### Characterization of antibody response induced by the PEDV-S mRNA vaccine in piglets

**Fig 3:**
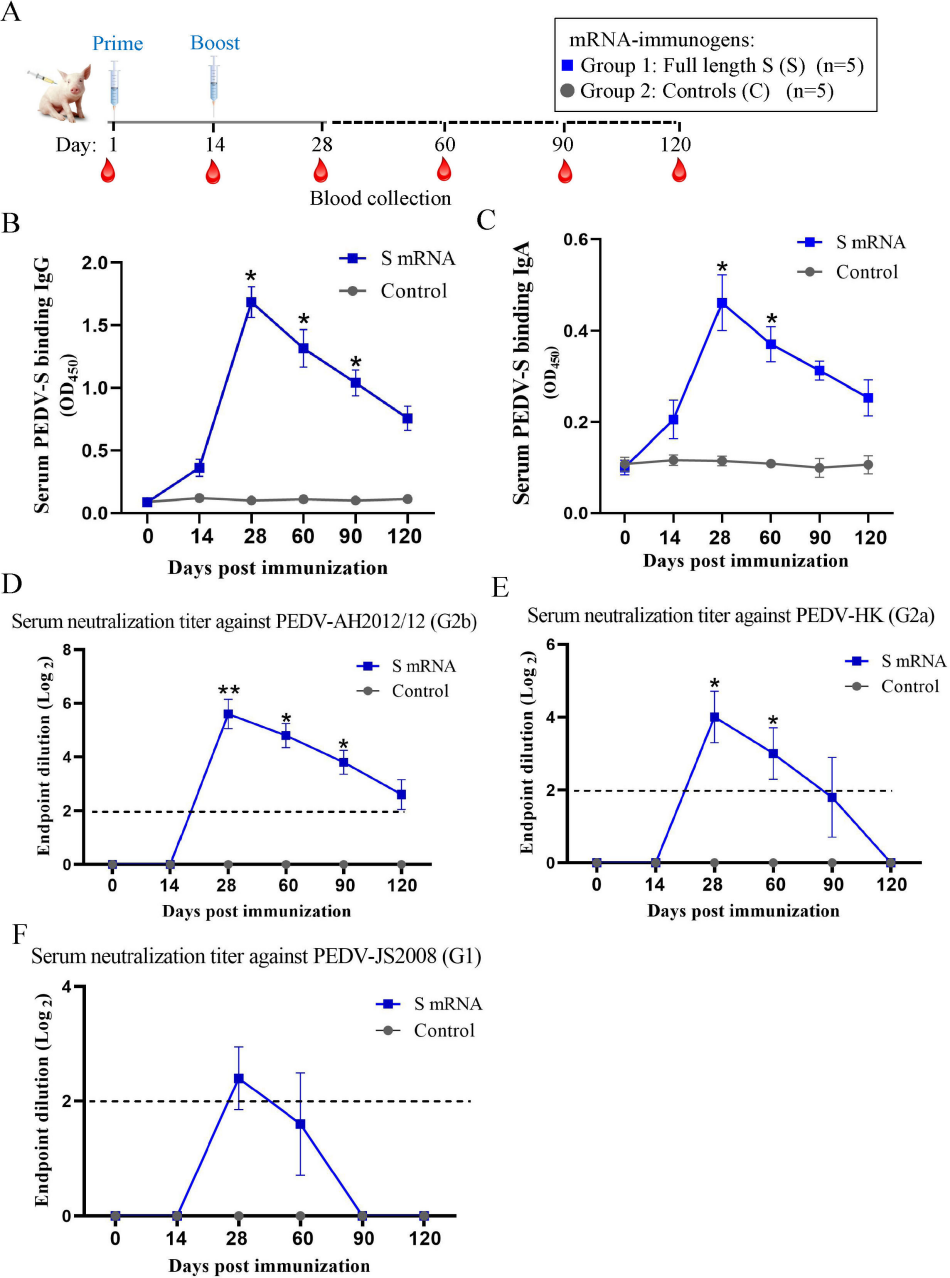
Characterization of antibody response induced by the PEDV-S mRNA vaccine in piglets. (**A**) Experimental design for the piglet vaccination. Neonatal piglets were immunized at two time points, day 1 and day 14 (after birth), with the full-length S mRNA (group 1) or PBS as a control (group 2). Serum samples were collected at 14, 28, 60, 90, and 120 d after the primary immunization, and the time course of PEDV S protein binding IgG (**B**) and IgA (**C**) antibodies titer was measured. (**D–F**) The serum-neutralizing antibodies against different PEDV strains over time were detected, including G2b strain AH2012/12 (**D**), G2a strain HK (**E**), and G1 strain JS2008 (**F**). The titers were expressed as the highest serum dilution that protected more than 50% of the cells from CPE. The dotted line indicates the lower limit of detection. Data are presented as the mean ± SD. All of the significant differences were relative to the control group. Significant differences are shown as *P < 0.05 and **P < 0.01.

We next evaluated the immunogenicity and efficacy of the PEDV-S mRNA vaccine in piglets, the natural host of PEDV. The study design is summarized in Fig. 3A. Briefly, two groups of piglets were inoculated with the S mRNA vaccine or PBS before receiving a booster dose at 14 dpv. The piglets experienced no adverse events after vaccination. Serum samples were collected before vaccination and at 13, 27, 60, 90, and 120 dpv to evaluate the levels of binding and neutralizing antibodies. The results showed that the titers of PEDV-S binding IgG (Fig. 3B) and IgA (Fig. 3C) antibodies were markedly increased after boosting with the S mRNA vaccine at 28 dpv. Although these antibody titers gradually decreased, the IgG antibody persisted relatively high, even at 120 dpv. The neutralizing antibodies appeared after the booster immunization. Similar to the results obtained from experiments in mice, the highest levels of neutralizing antibodies were generated against the homologous G2b strain (Fig. 3D). The neutralization ability of antibodies against the heterogeneous G2a strain was lower (Fig. 3E), while that of antibodies against the G1 strain was very weak (Fig. 3F). These data indicate that the S mRNA vaccine elicited a robust antibody response in piglets.

### Evaluation of PEDV-S-specific T cell responses in piglets

**Fig 4:**
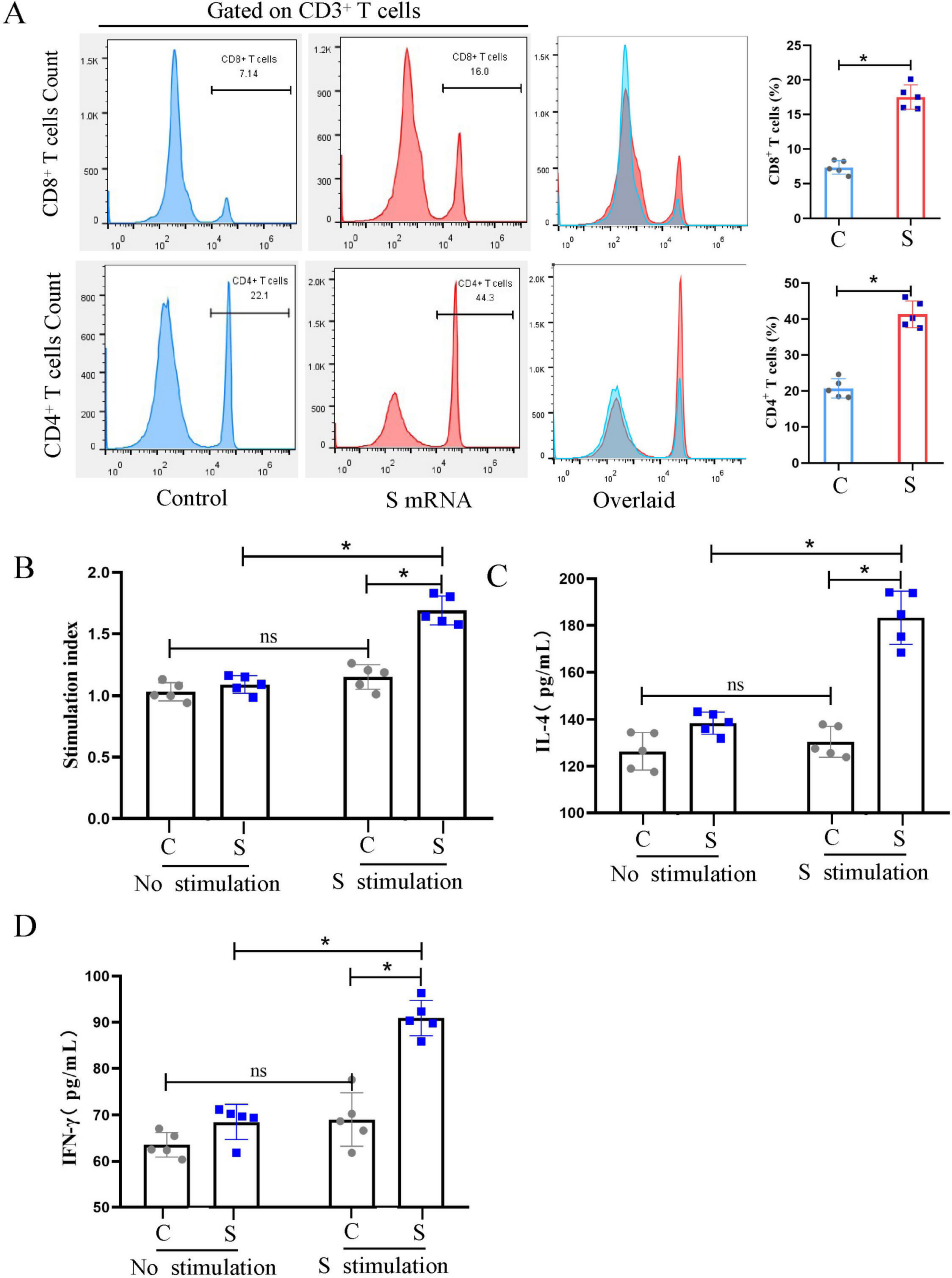
Evaluation of PEDV-S-specific T cell responses in piglets. Peripheral blood lymphocytes of piglets were isolated at 28 d post-immunization and submitted to assess cellular immune responses. (**A**) The frequency of CD3^+^CD4^+^ and CD3^+^ CD8^+^ T cells in piglets after immunization. The isolated lymphocytes of piglets were stained with the CD3, CD4, and CD8 fluorescent antibodies and then characterized by flow cytometry to enumerate CD4^+^ and CD8^+^ T cells per 1 × 10^5^ cells acquired. Data analysis was performed using FlowJo v10.7.1. (**B**) PEDV S-specific lymphocyte proliferation assay. The lymphocytes were stimulated with S protein for 72 h, and the proliferation rate was measured using a CCK-8 kit. The relative stimulation index was calculated as the ratio of the average OD value of antigen-stimulated wells to that of unstimulated wells. (**C and D**) IL-4 and IFN-γ secretion of lymphocytes after re-stimulation with S protein. The cell culture supernatant was collected for the IL-4 and IFN-γ detection by ELISA. Data are presented as the mean ± SD of five piglets per group. The asterisks in the figures indicate significant differences (*P < 0.05 and **P < 0.01; ns, not significant).

To evaluate the vaccine-induced cellular immune response in piglets, peripheral blood lymphocytes were isolated at 28 dpv and characterized by flow cytometry, focusing specifically on T lymphocyte subsets. We found that the percentages of CD3^+^CD4^+^ and CD3^+^CD8^+^ T cells in the S mRNA group were significantly higher than those in the PBS control group (Fig. 4A). To determine the magnitude of the antigen-specific T cell response, the peripheral blood lymphocytes were restimulated *in vitro* with the recombinant eukaryotic PEDV-S protein (10 µg/mL). We found that the stimulation index of the S mRNA immunization group was significantly higher than that of the control group (Fig. 4B). Again, the IFN-γ and IL-4 secretion of lymphocytes restimulated with the S protein was measured by ELISA. The mean IFN-γ and IL-4 levels produced by lymphocytes from the S-mRNA-immunized piglets were significantly higher than those produced by the lymphocytes of control piglets. These data demonstrate that the S mRNA vaccine induced PEDV-specific T cell responses in piglets.

### The PEDV-S mRNA vaccine protects against PEDV in actively immunized piglets

**Fig 5:**
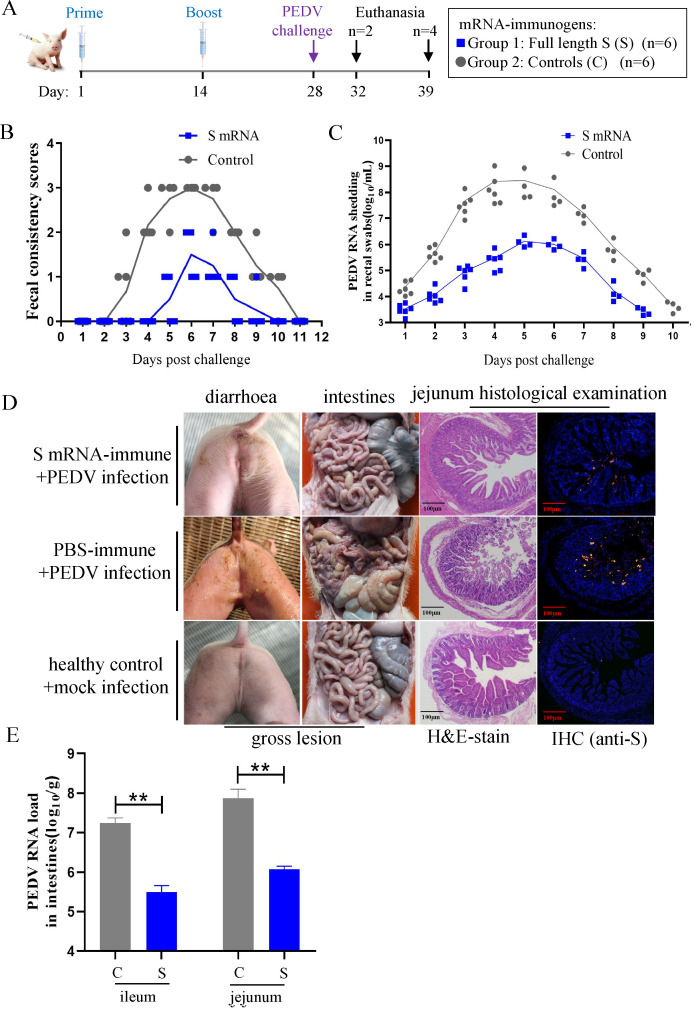
The PEDV-S mRNA vaccine protects against PEDV in actively immunized piglets. (**A**) Immunization and challenge schedule in piglets. Neonatal piglets were immunized with two doses of S mRNA vaccines, 14 d apart. The piglets were challenged with PEDV-AH2012/12 on day 28 and sacrificed at 4 d and 11 d post-challenge, respectively. (**B**) Diarrhea was assessed by scoring fecal consistency as follows: 0 being solid, 1 being pasty, 2 being semiliquid, and 3 being liquid. Piglets with scores of 2 or more were considered diarrheic. Each dot represents the score of an individual pig; each line indicates the mean scores of a group. (**C**) Viral shedding in rectal swabs was quantitated daily. Each symbol represents the titer of the PEDV N gene in 1 mL of rectal swab sample from an individual piglet collected daily. (**D**) Clinical diarrhea, and gross and histological lesions in intestine sections of piglets euthanized at 4 d post-challenge. The representative intestine sections are shown by post-mortem examination, H&E staining and immunohistochemistry assay. PEDV N was labeled with red fluorescence, and cell nuclei (blue) were stained with DAPI. Scale bar, 100 µm. (**E**) Virus load in the ileum and jejunum segments from different groups of piglets at 4 dpc. Data are presented as the mean ± SD. Significant differences are shown as *P < 0.05 and **P < 0.01 and attenuated vaccine group, scale bar, 50 µm.

Having verified the induction of neutralizing antibodies, we proceeded with the live viral challenge phase of the study (Fig. 5A). All the piglets were challenged with the PEDV AH2012/12 strain via the oral route. Their clinical signs, gross and histological lesions, and viral load were then evaluated. In addition, the incidence rate of diarrhea was assessed by scoring the fecal consistency of the challenged animals. Within 5 d post-challenge, no S mRNA vaccine-inoculated piglet had diarrhea (diarrhea scores < 2). Only two of the piglets immunized with the S mRNA vaccine had mild diarrhea (diarrhea scores = 2) lasting 1 or 2 d, and no cases of severe diarrhea were reported throughout the study. The piglets in the control group developed moderate diarrhea at 3 dpc, and most piglets in the control group had severe diarrhea (diarrhea scores = 3) for 4 d during the experiment. When the fecal PEDV RNA shedding profiles of the piglets were evaluated, we found that the piglets in the control group exhibited the highest level of viral shedding (10^9^ copies/mL) at 4 dpc. By contrast, the piglets in the S mRNA group exhibited relatively low levels of viral shedding (10^6^ copies/mL) at 5 dpc. In total, the piglets in the S mRNA group shed significantly less viral RNA from 2 to 9 dpc than the control piglets (Fig. 5C).

Notably, at 4 dpc, all piglets in the control group had mild to watery diarrhea, whereas all piglets in the S mRNA group had no obvious clinical signs. The typical diarrhea symptoms of piglets at 4 dpc are outlined in Fig. 5D. To compare the gross and histological lesions in the immunized and control animals, two piglets, randomly selected from each group, were killed and necropsied. No obvious gross lesions were observed in the intestinal tissues of piglets from the S mRNA group, while thin-walled, gas-distended intestines with minimal bleeding were observed in piglets from the control group. The pathological examination of tissue slices showed that the control piglets had blunted and fragmented villi and an atrophied jejunum, whereas the villi in the jejunum of S-mRNA-immunized piglets were predominantly normal. IF confirmed the presence of PEDV particles in the cytoplasm of epithelial cells comprising the atrophied villi of the control piglet jejunum; by contrast, only a few PEDV-positive cells were detected in the jejunum segments of the immunized piglets (Fig. 5D). We next measured the PEDV RNA loads in the jejunum and ileum of piglets and found that the viral RNA load was significantly higher in control than in the immunized piglets (Fig. 5E). These data demonstrate that the PEDV-S mRNA vaccine alleviated the clinical signs and pathological lesions, as well as lowered the viral load, in actively immunized piglets after PEDV challenge.

### Characterization of the antibody response elicited by the PEDV-S mRNA vaccine in sows

**Fig 6:**
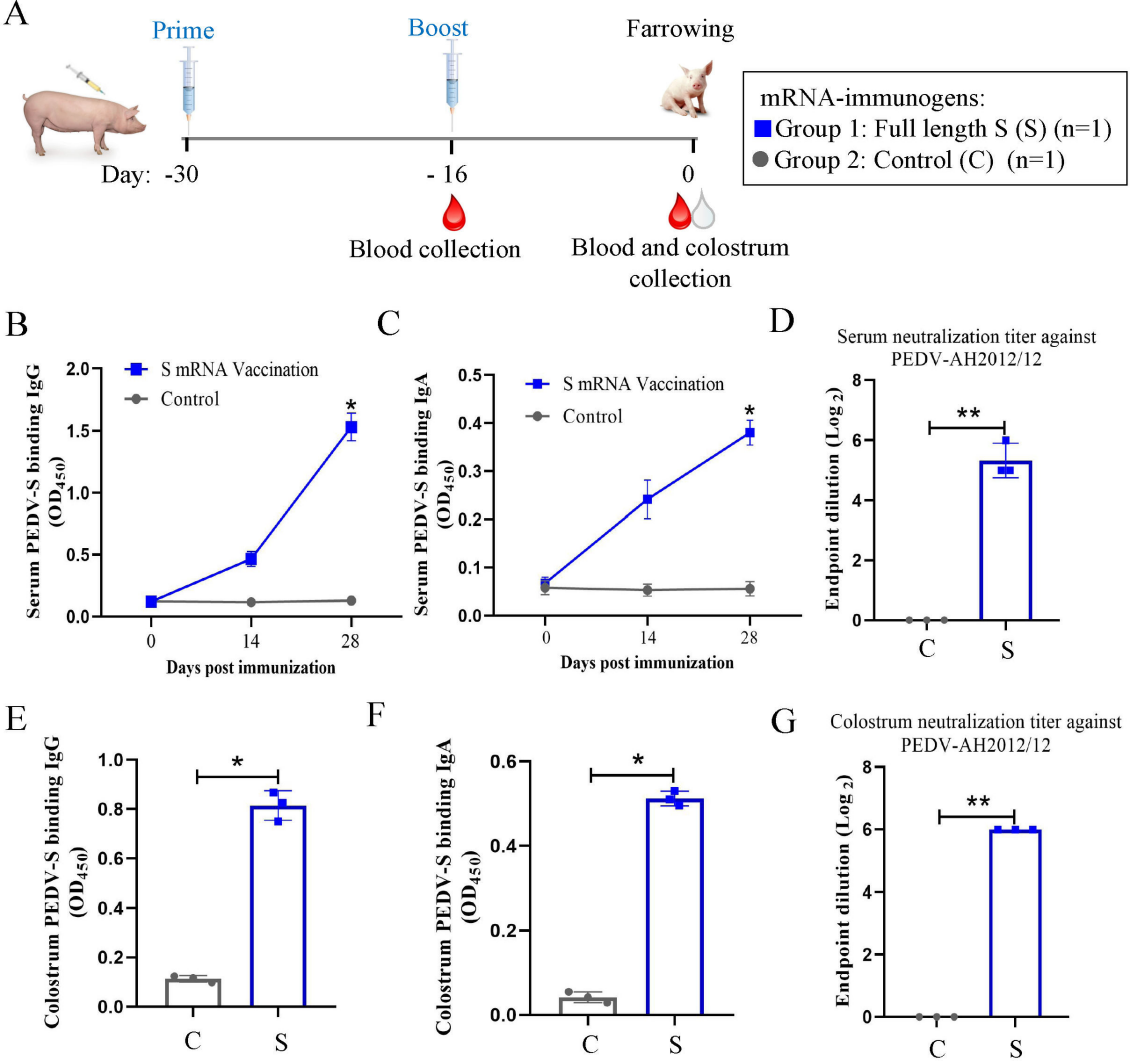
Characterization of the antibody response elicited by the PEDV-S mRNA vaccine in sows. (**A**) Immunization schedule in pregnant sows. Sows were immunized at two time points, 30 and 16 d before farrowing (set as day −30 and day −16), with the full-length S mRNA (group 1) or PBS as a control (group 2). Serum samples were collected at day −16 and the farrowing day (d 0), and colostrum was collected at the farrowing day. The levels of PEDV S protein binding IgG and IgA antibodies in serum (**B and C**) and colostrum (**E and F**) were measured. The neutralizing antibodies against PEDV-AH2012/12 strains in serum (**D**) and colostrum (**G**) were detected at day 0. All samples were subjected to three repeated detections.

In the passive immunization part of the study, we further evaluated the immunogenicity of the PEDV-S mRNA vaccine in pregnant sows. To this end, two pregnant sows were inoculated with the S mRNA vaccine or PBS; the vaccinated sow also received a booster dose at 14 dpv (Fig. 6A). The serum and colostrum were collected from the sows to detect the anti-PEDV antibody levels. The immunized sow did not experience any adverse events after vaccination. Similar to the results of the piglet vaccination experiments, the titers of PEDV-S binding IgG and IgA antibodies in serum (Fig. 6B and C) and colostrum (Fig. 6E and F) were markedly increased after boosting. The anti-PEDV-AH2012/12 neutralization ability of serum and colostrum antibodies was next determined. The neutralizing antibody titers of serum and colostrum reached 1:32 and 1:64, respectively, at 2 weeks after boosting (Fig. 6D and G).

### Passive protection against PEDV in suckling piglets is conferred by colostrum from immunized sows

**Fig 7:**
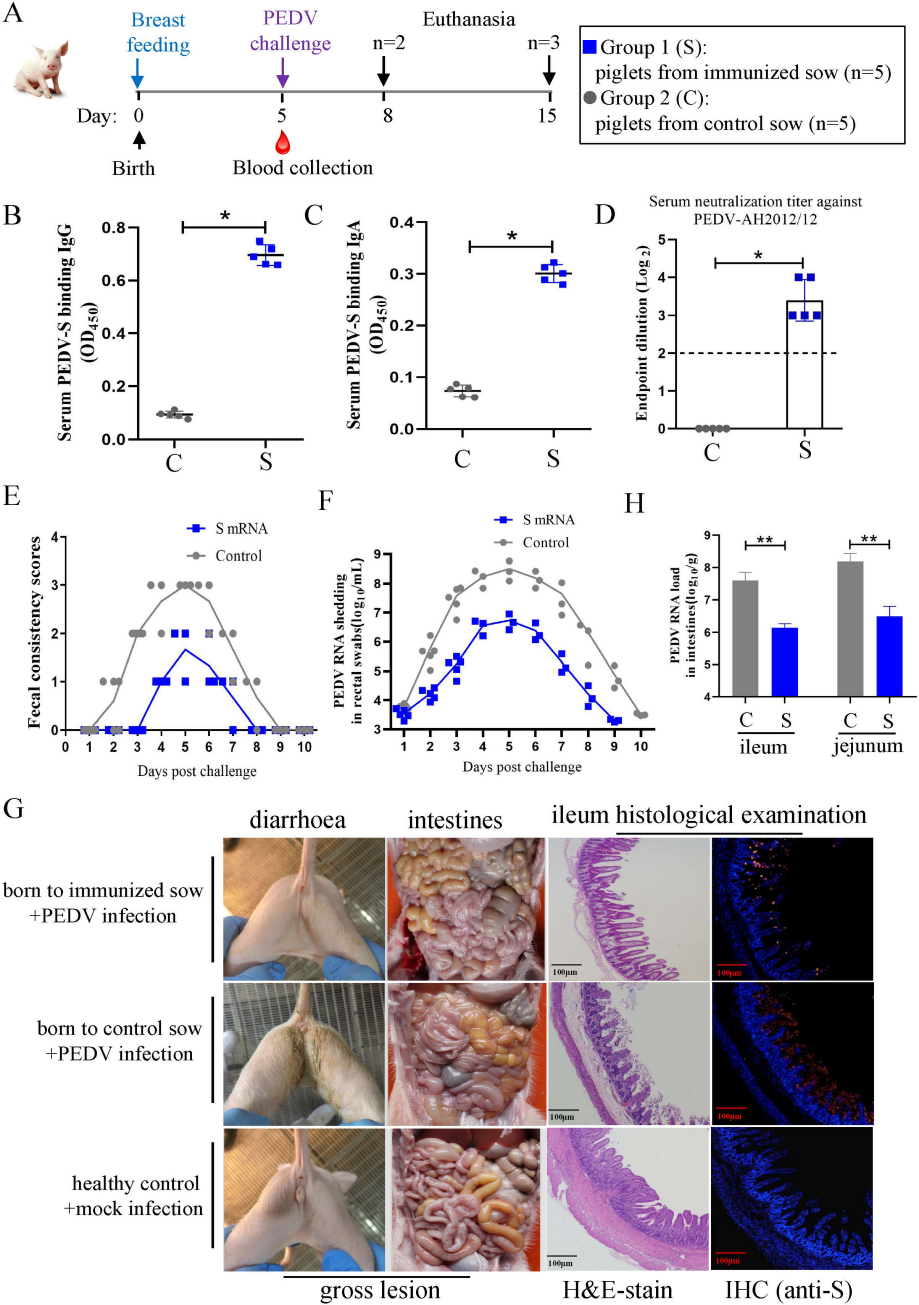
Passive protection against PEDV in suckling piglets is conferred by colostrum from immunized sows. (**A**) Challenge schedule in passively immunized piglets. After being breastfed for 5 d, neonatal piglets from each sow were challenged with PEDV AH2012/12 strain and sacrificed at 3 d and 10 d post-challenge, respectively. (B–D) The level of passively transferred antibodies in piglet sera. Serum samples were collected from each piglet at day 5 for detection of PEDV S protein binding IgG, IgA, and the neutralization antibodies. The dotted line indicates the lower detection limit. (**E**) Diarrhea was assessed by scoring fecal consistency. Piglets with scores of 2 or more were considered diarrheic. (**F**) Viral shedding in rectal swabs was quantitated daily. Each symbol represents the titer of the PEDV N gene in 1 mL of rectal swab sample from an individual piglet collected daily. (**G**) Clinical diarrhea, gross, and histological lesions in intestine sections of piglets euthanized at 3 d post-challenge. The representative intestine sections are shown by post-mortem examination, H&E staining, and immunohistochemistry assay. PEDV N was labeled with red fluorescence, and cell nuclei (blue) were stained with DAPI. Scale bar, 100 µm. (**H**) Virus load in the ileum and jejunum segments from different groups of piglets at 4 dpc. Data are presented as the mean ± SD. Significant differences are shown as *P < 0.05 and **P < 0.01.

The ability of the S mRNA vaccine to confer passive immunity and protection was next investigated in suckling piglets. After suckling for 5 d, the piglets born to immunized and control sow were challenged with PEDV (Fig. 7A). The levels of passively transferred antibodies were detected in the piglet serum. PEDV-S-binding IgG (Fig. 7B) and IgA (Fig. 7C) antibodies, as well as neutralizing antibodies (Fig. 7D) against PEDV-AH2012/12, were detected in the sera of piglets born to the immunized sow but not in those born to the control sow, demonstrating the transfer of antibodies through colostrum. All piglets born to the control sow had moderate to watery diarrhea starting at 3 dpc, which lasted for 5 d. Meanwhile, only two piglets born to the immunized sow started experiencing mild diarrhea at 6 dpc, which lasted for 2 d (Fig. 7E). Piglets born to the immunized sow had a significantly lower level of PEDV RNA in their feces in comparison with that of the control piglets (Fig. 7F). To compare the gross and histological lesions between the offspring of the immunized and the control sow, two piglets, randomly selected from each litter, were killed and necropsied at 3 dpc. No obvious gross lesions were observed in the intestinal tissues of the piglets from the immunized sow, whereas thin-walled and gas-distended intestines were observed in the piglets from the control sow. Pathological examination revealed no obvious histopathological changes in the ileum of piglets born to the immunized sow. However, the ileum of piglets born to the control sow exhibited significant histopathological changes, including villous blunting and atrophy, vacuolation, loss of intestinal crypts, and villous architecture. IF showed PEDV accumulation in the atrophied villi of the control piglet ileum segments, while only a small fraction of PEDV-positive cells was detected in the ileum segments of the immunized piglets (Fig. 7G). We also measured the PEDV RNA loads in the jejunum and ileum of piglets, and the results showed that the viral load in the control piglets was significantly higher than that in the immunized piglets (Fig. 7H). Together, these results demonstrate that the PEDV-S mRNA vaccine endowed newborn piglets with effective passive immunity, which protected them against the PEDV challenge.

## DISCUSSION

PEDV is classified as an emerging and re-emerging *α*-coronavirus, which poses a significant threat to piglets and causes large economic losses to the global swine industry. Thus, the development of protective vaccines against PEDV remains a top priority. The success of mRNA-LNPs as COVID-19 vaccines heralded a new era of vaccine development, because mRNA-based vaccines have additional beneficial features over other vaccine platforms (29). Moreover, mRNA vaccine antigen coding sequence can be rapidly designed and produced; this flexibility of the mRNA platform can be highly advantageous when immunogens need to be rapidly updated in response to emerging pathogenic variants (30). Despite such advantages, no studies have been published on PEDV mRNA vaccines. Here, we provided a detailed evaluation of a PEDV mRNA vaccine expressing the S protein in piglets. The data presented in this study have demonstrated the potential of mRNA vaccines in conferring protection against PEDV infection. Based on these encouraging preliminary findings, efforts should be made to develop a PEDV mRNA vaccine for use in the real world.

The PEDV S glycoprotein comprises of the S1 and S2 domains, which play important roles in viral attachment to the host cell and virus-cell membrane fusion. The S1 domain mediates viral entry and attachment and induces neutralizing antibodies, while the S2 domain is responsible for membrane fusion. The COE and NTD in the S1 region have been reported to act as a neutralizing epitope and a potential co-receptor binding area for PEDV, respectively (31, 32). Thus, the full-length S, S1, and COE of PEDV are the primary targets for vaccine development. In recent years, different forms of S, S1, and COE, expressed in various expression systems (e.g., mammalian cells, *Escherichia coli*, baculovirus, *Bacillus subtilis*, adenovirus, and *Lactobacillus*), have been used as immunogens to develop PEDV vaccines (33–37). Our previous study found that a subunit vaccine based on the PEDV Sm, composed of the NTD, COE, and several linear neutralizing epitopes, induced an effective immune response against PEDV (26). In this study, we designed two mRNA vaccines, encoding either the full-length S or the Sm protein, and compared their immunogenicity in mice. The data revealed that the full-length S mRNA vaccine induced higher levels of neutralizing antibodies than the Sm mRNA vaccine. Thus, the full-length S protein may be a better target for mRNA vaccine development than the Sm version because the S protein may have undiscovered neutralizing sites.

The efficacy of mRNA vaccines is due to their ability to induce both humoral and cellular immunity. Our results showed that although the PEDV S mRNA vaccine induced general levels of neutralizing antibodies, it provided a reasonable level of immune protection against PEDV, suggesting that the vaccine efficacy was likely due to a combination of neutralizing antibodies and other immunologic factors, such as the cellular immune response. Indeed, we demonstrated that the PEDV S mRNA vaccine simultaneously activated both CD4^+^ T cells and CD8^+^ T cells. However, this study did not perform an in-depth characterization of cellular immunity induced by the mRNA vaccine and requires further investigation.

Broad spectrum activity is a key factor in the design of an effective vaccine. The flexibility of the mRNA vaccine platform is conducive to the research and development of broad-spectrum vaccines. It has been reported that wide-spectrum mRNA vaccines for COVID-19, influenza viruses, and rabies viruses show good immune results (38–42). Based on their *S* gene sequences, PEDV isolates can be divided into the G1a, G1b, G2a, and G2b subtypes. Both G1 and G2 PEDV strains are circulating among pigs in different countries. Moreover, differences in pathogenicity exist between representative isolates of different genogroups, with G2b isolates being typically more pathogenic than the other isolates (43, 44). Partial to no cross-protection between different isolates has been demonstrated, which may be one of the reasons for the poor efficacy of PEDV vaccines (45–47). In the present study, we developed an mRNA vaccine encoding the S protein of the PEDV G2b strain AH2012/12 and determined the neutralizing activity of the antibodies in the serum of immunized mice and piglets. The results showed that the highest level of neutralizing antibodies was elicited against the homologous G2b strain, while a lower amount of neutralizing antibodies targeted the heterogeneous G2a strain. The neutralizing ability against the G1 strain was very weak. The partial cross-neutralizing ability of the PEDV S mRNA vaccine against G2a was observed; however, this was only weak against the G1 strain. Therefore, future endeavors should focus on developing a PEDV mRNA vaccine with greater cross-neutralizing ability.

Passive immunity is critical for the protection of neonatal piglets against PEDV. Passive transfer of antibodies through colostrum and milk provides immediate protection to piglets at this vulnerable stage of life. The colostrum of sows contains large amounts of IgG and IgA. In sucking neonatal piglets, the IgG is transported across neonatal enterocytes. It enters the neonatal bloodstream to provide systemic protection. At the same time, the IgA could be transferred across the “open” gut to the general circulation during the first few days of life and then mainly persists in the lumen of the neonatal intestine to provide local protection (48, 49). Therefore, a vaccine that elicits systemic IgG and local IgA responses in sows should be developed. In this study, we succeeded in inducing PEDV-specific IgG and IgA in the serum and colostrum of S-mRNA-immunized sows. IgG and IgA were secreted in the colostrum and transferred to suckling neonatal piglets.

In conclusion, we designed two mRNA vaccines expressing the PEDV S glycoprotein and investigated their immunogenicity and ability to protect piglets from PEDV infection. We demonstrated that the full-length PEDV S mRNA vaccine induced robust PEDV-specific humoral and cellular immune responses and conferred protection against PEDV infection to actively and passively immunized piglets. Thus, S mRNA is a promising platform for developing a preventive PEDV vaccine.
